# Metagenomic approach revealed the mobility and co-occurrence of antibiotic resistomes between non-intensive aquaculture environment and human

**DOI:** 10.1186/s40168-024-01824-x

**Published:** 2024-06-14

**Authors:** Li Tian, Guimei Fang, Guijie Li, Liguan Li, Tong Zhang, Yanping Mao

**Affiliations:** 1 College of Chemistry and Environmental Engineering, Shenzhen University, Shenzhen, 518071 Guangdong China; 2 The University of Hong Kong Shenzhen Institute of Research and Innovation, HKU SIRI, Shenzhen, Guangdong 518057 China; 3 Department of Civil Engineering, Environmental Microbiome Engineering and Biotechnology Laboratory, Centre for Environmental Engineering Research, The University of Hong Kong, Hong Kong SAR, China

**Keywords:** Aquaculture, Metagenome, Resistome, ARG mobility, One Health

## Abstract

**Background:**

Aquaculture is an important food source worldwide. The extensive use of antibiotics in intensive large-scale farms has resulted in resistance development. Non-intensive aquaculture is another aquatic feeding model that is conducive to ecological protection and closely related to the natural environment. However, the transmission of resistomes in non-intensive aquaculture has not been well characterized. Moreover, the influence of aquaculture resistomes on human health needs to be further understood. Here, metagenomic approach was employed to identify the mobility of aquaculture resistomes and estimate the potential risks to human health.

**Results:**

The results demonstrated that antibiotic resistance genes (ARGs) were widely present in non-intensive aquaculture systems and the multidrug type was most abundant accounting for 34%. ARGs of non-intensive aquaculture environments were mainly shaped by microbial communities accounting for 51%. Seventy-seven genera and 36 mobile genetic elements (MGEs) were significantly associated with 23 ARG types (*p* < 0.05) according to network analysis. Six ARGs were defined as core ARGs (top 3% most abundant with occurrence frequency > 80%) which occupied 40% of ARG abundance in fish gut samples. Seventy-one ARG-carrying contigs were identified and 75% of them carried MGEs simultaneously. The *qacEdelta*1 and *sul*1 formed a stable combination and were detected simultaneously in aquaculture environments and humans. Additionally, 475 high-quality metagenomic-assembled genomes (MAGs) were recovered and 81 MAGs carried ARGs. The multidrug and bacitracin resistance genes were the most abundant ARG types carried by MAGs. Strikingly, *Fusobacterium*_A (opportunistic human pathogen) carrying ARGs and MGEs were identified in both the aquaculture system and human guts, which indicated the potential risks of ARG transfer.

**Conclusions:**

The mobility and pathogenicity of aquaculture resistomes were explored by a metagenomic approach. Given the observed co-occurrence of resistomes between the aquaculture environment and human, more stringent regulation of resistomes in non-intensive aquaculture systems may be required.

Video Abstract

**Supplementary Information:**

The online version contains supplementary material available at 10.1186/s40168-024-01824-x.

## Introduction

Aquaculture occupies a significant portion of the global food supply, and the growing consumption of aquatic animal protein surpasses that of all other animal proteins combined. Asia represented 69% of worldwide aquaculture production, with China alone accounting for 35% [1, 2]. The demand for aquaculture animals is expected to increase by 27% by 2030 [3]. At present, in response to the increasing demand for aquatic products, the typical approach is the intensification of aquaculture production or extensive use of antibiotics [4, 5]. However, the extensive use of antibiotics will lead to the emergence and enrichment of antibiotic resistance bacteria and antibiotic resistance genes (ARGs) [6, 7]. Antibiotic resistance in aquaculture might further lead to the prevalence of aquatic animals increasing and aquaculture production reducing [1, 8]. In addition, ARGs could be transferred through the food chain into humans, posing potential health risks [1, 9].

Previous studies have found that high concentrations of antibiotics were the main driving factors of the development and transfer of resistomes in aquaculture environments [10, 11]. Even though the antibiotics use was restricted, antibiotic residues in livestock manure or irrigation water could form a certain selective pressure promoting horizontal gene transfer (HGT) through mobile genetic elements (MGEs) [12–14]. However, most previous studies focused on intensive aquaculture environments, where antibiotics were often present at high concentrations. Traditional intensive aquaculture often demands a large number of cultured animals in a confined space, which will promote the transfer of resistome and parasite infection, resulting in a reduction of aquaculture production [15]. Non-intensive aquaculture, which is based on privately owned fishponds with much less antibiotic use than intensive aquaculture, is the most common form of aquaculture in southern China [16]. Non-intensive aquaculture provides more space and avoids overuse of feed. The relatively extensive operation mode of non-intensive aquaculture is more likely to produce environmental interferences [17]. However, the influencing factors of the emergence and transfer of resistomes in non-intensive aquaculture environments are not yet clear. Although some ARGs have been found in non-intensive aquaculture environments [18–20], their potential mobility and pathogenicity have not been well evaluated.

Under the “One Health” concept, the transfer of resistomes between the environment, animals, and humans has received extensive attention [8, 21, 22]. Previous researches on the resistome transfer often focused on limited environmental components, such as animal waste and surrounding soil or water [23–25], fishpond sediment and water [12, 16, 19, 26], activated sludge, and influent or effluent from wastewater treatment plants [27–29]. There is still very little scientific understanding of resistome development in an integrated system including humans, animals, and the environment. It is thus necessary to explore the pattern of ARG transfer between complex environmental system and humans to deepen the understanding of potential health risks.

In this study, we conducted comprehensive sampling (including the chicken gut, fish gut, fishpond sediment, and water) from a non-intensive aquaculture farm and collected local human gut metagenomic data from a public database. A metagenomic approach was employed to characterize the distribution and mobility of resistomes. The results revealed the potential transfer risk of various ARGs between aquaculture environment and human. The pathogens carrying multiple ARGs shared with the aquaculture system and human gut were identified. Our study emphasizes the transfer potential as well as the health risks of aquaculture resistomes and provides a scientific basis for controlling resistomes within the One Health concept.

## Materials and methods

### Sampling and sequencing

Sampling activities were conducted at a non-intensive aquaculture farm in southern China (113.33 E, 22.10 N). The fish and chickens were raised on the farm without antibiotics. The chickens were fed with natural grains free of antibiotics, the composition of the fish feed could be found in Table S1, and the concentrations of antibiotics and metals in fishpond water were measured (Table S2). Chicken gut samples were collected from chickens at different growth stages with different weights. The fish gut samples of different species, including carp, tilapia, grass carp, bighead carp, and crucian carp, were collected. The fish gut specimens were squeezed out, and the contents were collected carefully to avoid contamination of the intestinal tissue [30]. Fresh chicken droppings were collected from the coop as chicken gut samples [31]. The sediment and water samples were collected at the four corners of the fishponds and then mixed separately. For each water sample, 300 mL of water was filtered using 0.22-µm microporous membranes (Millipore, MA, USA) to enrich microorganisms. All samples were stored at −80 ℃ until DNA extraction. Finally, 35 samples were collected, including 6 chicken guts, 17 fish guts, 8 sediments, and 4 water samples. The detailed sample information can be found in Table S3.

E.Z.N.A.® Water DNA Kit (Omega Bio-Tek, USA) was used for DNA extraction from water samples, and FastDNA® SPIN Kit for Soil (MP Biomedicals, USA) was used for DNA extraction of the other samples according to the manufacturers’ protocols. The extracted DNA was used to construct the library with an insert size of 350 bp and sequenced using the paired-end (PE) 150 bp strategy on the Illumina NovaSeq platform by Novogene company (Beijing, China). All the metagenomic sequencing raw data have been submitted to the Sequence Read Archive (SRA) of NCBI (PRJNA988937). The “aquaculture system samples” in downstream data analysis refers to the collection of all chicken gut, fish gut, sediment, and water samples.

To further analyze the sharing of resistomes between aquaculture system and human, 30 metagenomic data sets about the local human gut (Guangdong Province, China) were downloaded from the NCBI SRA database (Table S4). According to the reference, the volunteers did not receive any antibiotic treatment for 2 months before sampling [32]. Metagenomic data sets with size > 4.5 Gb were selected, and a total of 311.6 Gb of data was downloaded. The raw data was filtered according to the human genome reference (hg18) to eliminate human host DNA before subsequent analysis [32].

### Identification and quantification of bacteria, ARGs, and MGEs

All raw reads were quality-filtered using fastp (v0.20.1) [33] to remove sequences with a quality value < 20 or ambiguous nucleotides > 10. After quality filtering, clean reads were used for taxonomic annotation and quantification by Kraken 2 (v2.0.7) [34] and Bracken (v2.0) [35]. ARGs-OAP (v3.0) [36, 37] was used for identification and quantification of ARGs. Clean reads were aligned with the structured ARG database (SARG v3.0) by BLAST + (v2.12.0) (similarity ≥ 80%, *e *value ≤ 1e − 7 and query coverage ≥ 75%). Finally, the abundance of ARGs was indicated as a “copy of ARG per copy of 16S rRNA gene” based on ARGs-OAP [38, 39]. The analysis of MGEs followed the similar procedure as ARGs, and the SARG database was replaced with the MGE database constructed in the previous reference [40]. This MGE database contains 278 distinct gene annotations and at least 2000 unique sequences.

### Metagenomic assembly and binning

After quality filtering, clean reads were assembled using SPAdes (v3.15.3) [41] with the parameters “–meta”. The quality of contigs was evaluated by quast (v5.0.2) [42], and contigs longer than 1000 bp were used for subsequent analysis. Kraken2 (v2.0.7) and Bracken (v2.0) were employed for identifying the taxonomy of contigs. Prodigal [43] (v2.6.3) was used to predict open reading frames (ORFs) on contigs. ARGs and MGEs were searched among the predicted ORFs using BLAST + (v2.12.0) against the database of ARGs and MGEs (refer to 2.2 for database details, *e* value ≤ 1e − 5, identity ≥ 60%). The ARG-carrying contig (ACC) will be used for the subsequent analysis about the co-occurrence of ARGs and MGEs. The opportunistic pathogenic ACC was identified according to a published list of opportunistic pathogenic species [44].

The co-assembly results of each sample type (chicken gut, fish gut, sediment, water, and human gut) were imported to MetaWRAP (v1.3.2) [45] to recover metagenomic-assembled genomes (MAGs), and the *bin_refinement* module was performed to improve the quality of MAGs. The completeness and contamination of the recovered MAGs were estimated using CheckM (v1.2.0) [46] with default parameters (completeness > 50% and contamination < 10%). Only high-quality MAGs (completeness—5*contamination ≥ 50%) were retained for downstream analysis. The recovered MAGs were dereplicated using dRep (v3.4.0) [47], and the MAGs with average nucleotide identity (ANI) ≥ 95% were considered to be the same species [48, 49]. The *Quant_bin* module (default parameters) from MetaWRAP was used to calculate the relative abundance of non-redundant MAGs, and genome copies per million reads (GPMR) was used as the abundance unit. GTDB-Tk (v1.4.1) [50] was used to assign taxonomic classifications to the MAGs, and FastTree [51] was employed to perform phylogenetic analyses and construct phylogenetic trees, which were then visualized by iTOL (https://itol.embl.de).

### Co‑occurrence analysis of ARGs and MGEs

To investigate the transfer potential of ARGs between the aquaculture system and human, gene arrangements were deciphered on ACC. The different ARG-MGE combinations that existed simultaneously between different sample groups were visualized by R package *gggenes*. To further analyze the co-occurrence of ARG-MGE on contigs, two indices introduced by Li et al. [52] were employed to measure their co-occurrence (incidence of encountering and average minimum distance). Briefly, the average minimum distance was calculated by adding the distance from each ARG to the nearest MGE and then dividing it by the total number of ARGs. The number of MGEs found within a certain range per ARG was counted, and the average number of MGEs found in each contig was used to determine the incidence of ARGs encountering MGEs.

### The model and method of ARG source tracking

SourceTracker (v1.0) was a useful classification tool based on Bayesian and machine learning [53]. It has been verified to be effective in predicting ARG sources from complex environmental samples [54, 55]. Here, SourceTracker was used for tracking human gut ARGs from various environments, including soil, river, estuary, activated sludge, and non-intensive aquaculture system. The different environment data were collected from the NCBI SRA database (Table S5) and processed in accordance with the above methods (refer to 2.2). The abundances of ARG subtypes were used as SourceTracker input and the following parameters were set: burnin = 100, nrestarts = 10, delay = 10, *α* = 0.001, *β* = 0.01, and rarefaction_depth = 1000. The leave-one-out strategy was used to evaluate the performance of the model and the suitability of source settings as previous research described [56].

### Statistical analysis and visualization

All statistical analyses were performed in R (v4.1.3) software. Data sets were normalized before analyses, and the results with *p* < 0.05 by *t* test were considered as statistically significant. The differences in ARG composition and abundance between different sample groups were revealed by principal coordinate analysis (PCoA). The visualization of network analysis was performed by Gephi (0.9.2) [57]. Variation partitioning analysis (VPA) was used to explore the effects of microbial communities and MGEs on ARGs in aquaculture systems. TBtools [58] was used to make heatmaps. These statistical analyses were performed by *vegan* package in R, and the *ggplot2* package was used for result visualizations.

## Results

### ARG profile in the aquaculture environment

**Fig. 1:**
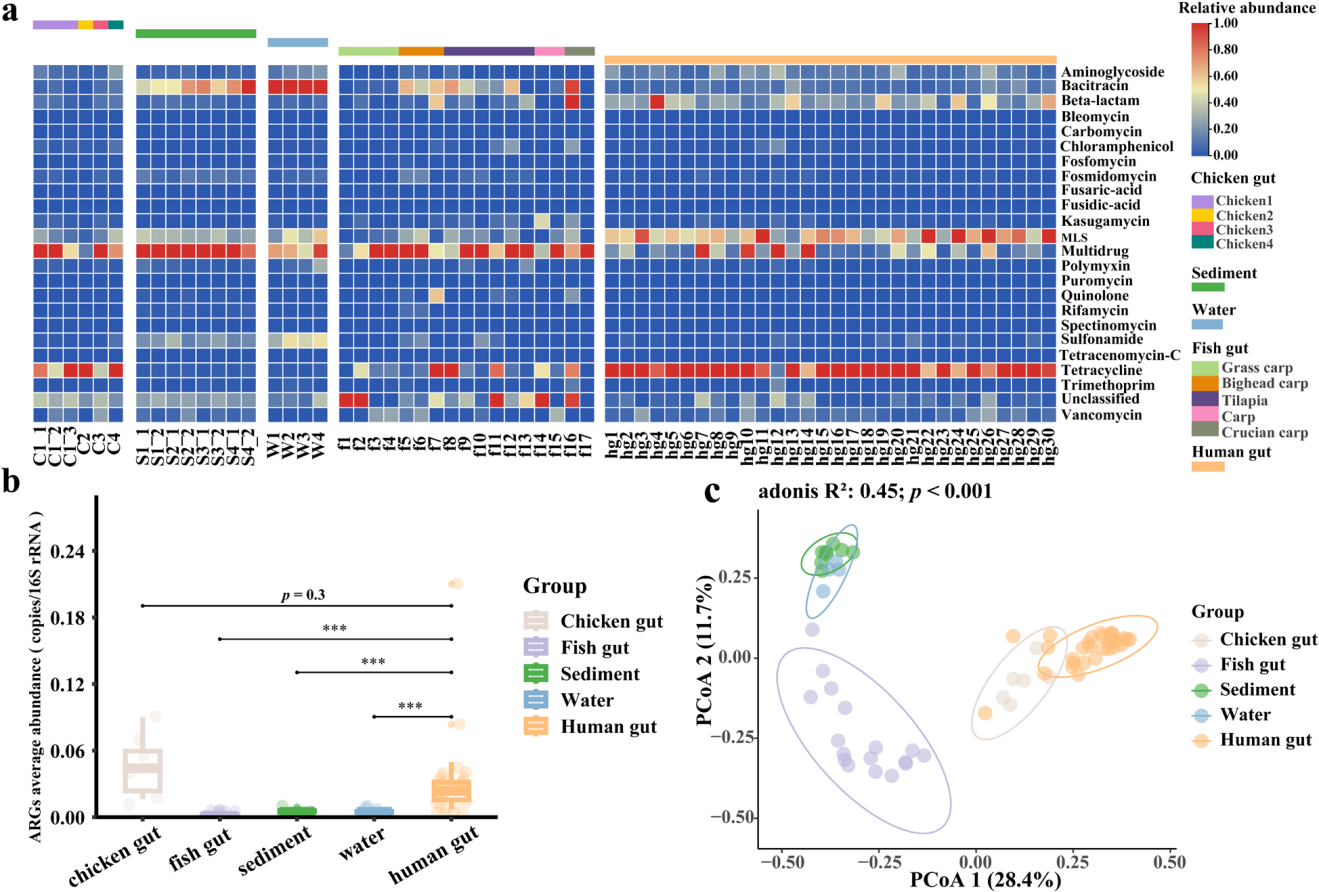
ARG composition and abundance profile across samples. **a** Heatmap of ARG relative abundance (log-transformed). Different color blocks at the top represent different sample groups. **b** Box plot of ARG abundance in different sample groups. Significant difference between groups is indicated by asterisk with *t* test (***p < 0.001). **c** PCoA shows the sample clustering based on ARG abundance profile (95% confidence interval)

A total of 22 types and 576 subtypes of ARGs were detected, and the distribution of ARGs was slightly different among different sample groups (Fig. 1a). Overall, multidrug resistance genes (MDR) were relatively abundant in aquaculture system samples, accounting for about 34% of the total ARG abundance. In contrast, macrolide-lincosamide-streptogramin (MLS) and tetracycline resistance genes were more abundant in human gut samples, accounting for about 24% and 41% of the total ARG abundance. MDR and tetracycline resistance genes were relatively abundant ARG types in chicken gut counting for 30% and 35%. The abundances of MDR and bacitracin resistance genes were relatively high in sediments and water, counting for 31% and 27%. Interestingly, we observed that MDR were consistently the most abundant ARGs across the five fish species of different feeding habits (i.e., grass carp, bighead carp, tilapia, carp, and crucian carp).

The average abundance of all ARG types detected in chicken gut, fish gut, sediment, water, and human gut were 0.050 ± 0.03, 0.002 ± 0.001, 0.006 ± 0.002, 0.005 ± 0.0009, and 0.030 ± 0.04 copies of ARG per copy of 16S rRNA gene, respectively (the data were expressed as means ± standard deviation). Compared with the other sample groups, the average abundances of ARGs were higher in the chicken gut and the human gut. The abundance of ARGs in the human gut was significantly different from fish gut, sediment, and water (*t* test, *p* < 0.001) (Fig. 1b). In order to compare the composition of ARGs in different sample groups, PCoA based on Bray–Curtis distance was performed and obvious clustering between the sample groups was observed (*p* < 0.01) (Fig. 1c). The clustering of water and sediment indicated that their composition of ARGs was not significantly different, while the fish gut samples were clustered separately.

### The identification of aquaculture core ARGs

**Fig. 2:**
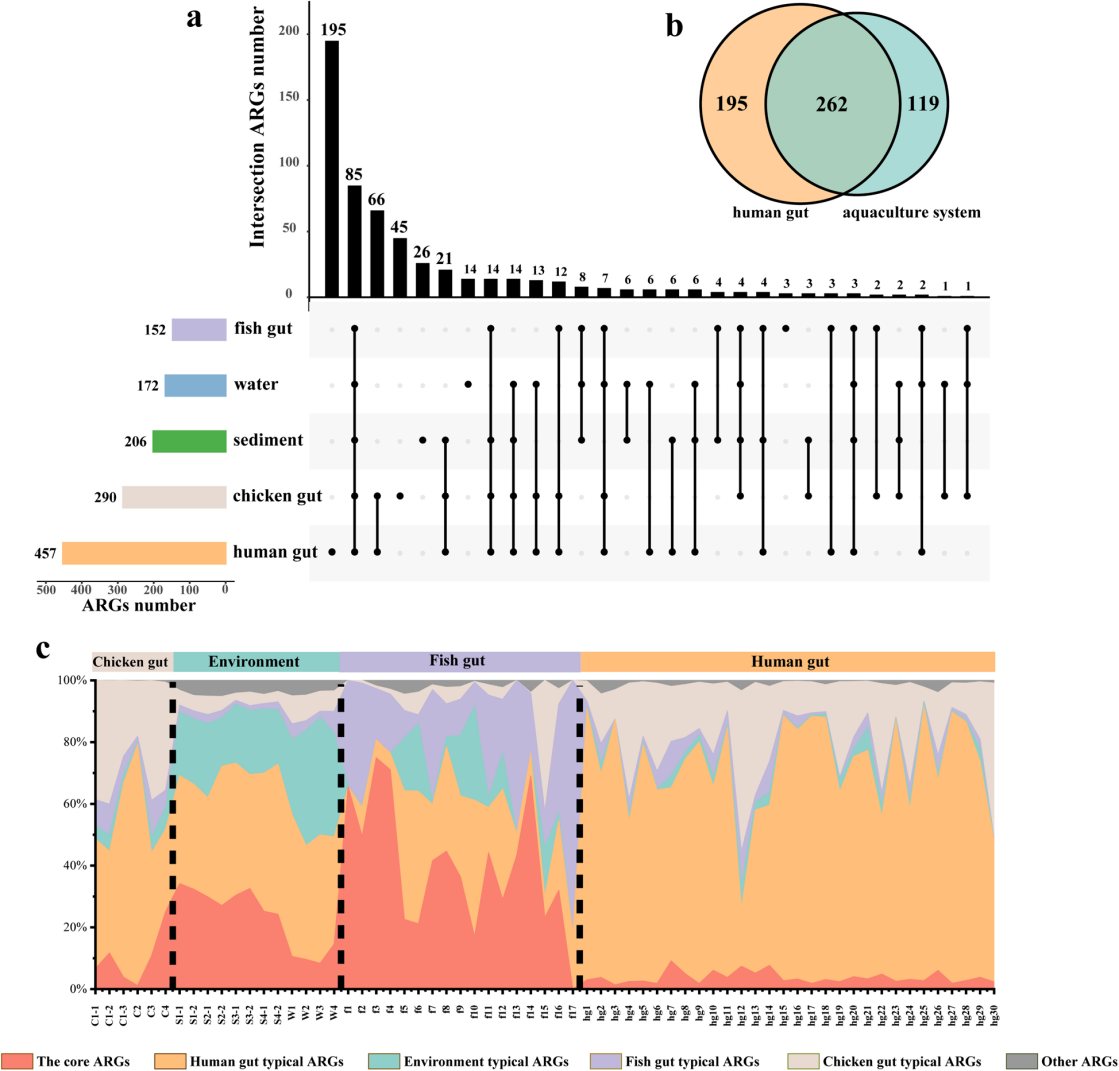
The core ARGs in aquaculture system. **a** UpSet diagram shows the number of ARGs shared and unique between different sample groups. Solid black points indicate ARG occurrence in the sample group, and points linked by lines indicate ARGs shared by different sample groups. The bar chart on the top shows the number of ARG unique or shared by sample groups, and the bar chart on the left shows the total number of detected ARG subtypes. **b** The Venn diagram shows the number of ARGs shared by the human gut and the aquaculture system. **c** The percent stacked histogram shows the composition of typical and core ARGs (indicated by different colors). Human gut and environmental typical ARGs defined as ARGs detected in all human gut and environment samples (fishpond sediment and water samples), respectively. Fish gut typical ARGs defined as ARGs detected in 24% fish gut samples, and chicken gut typical ARGs defined as ARGs detected in 50% chicken gut samples

In order to better understand the connection of ARGs between aquaculture and human gut, the common and distinct ARGs between various sample groups were identified. Four hundred fifty-seven ARG subtypes were detected in the human gut, and 195 of them were uniquely carried (Fig. 2a). There were 262 ARG subtypes detected simultaneously in the human gut and aquaculture system (Fig. 2b), accounting for 57% of the ARGs carried in the human gut. It is worth noting that 85 ARG subtypes were present in all five sample groups, and 66 ARG subtypes were carried by both human gut and chicken gut (Fig. 2a), indicating certain overlap of ARGs between aquaculture system and human.

The core ARGs of the aquaculture system were identified according to the ARG occurrence frequency. The negative correlation between the subtype number and occurrence frequency of detected ARGs (*R*^2^ = 0.892) demonstrated that the number of persistent ARGs in the aquaculture system was limited (Fig. S1). Notably, only 6 ARG subtypes had occurrence frequency greater than 80% (described as core ARGs), and 229 ARG subtypes had occurrence frequency less than 20% (described as transient ARGs). In addition, the average abundance of core ARGs was 5.95 × 10^–3^ copies of ARG per copy of 16S rRNA gene, while the average abundance of the transient ARGs was only 7.31 × 10^–5^ copies of ARG per copy of 16S rRNA gene which was much lower than core ARGs. Overall, the core ARGs with low diversity but high abundance were found in the aquaculture system. These core ARGs accounted for 29% ± 20% and 4% ± 2% of the ARGs composition in aquaculture systems and human gut, respectively (Fig. 2c). It is worth noting that the core ARGs occupied a substantial fraction of ARG abundance in fish gut samples (40% ± 21%).

### The influencing factors of ARGs in the aquaculture system

**Fig. 3:**
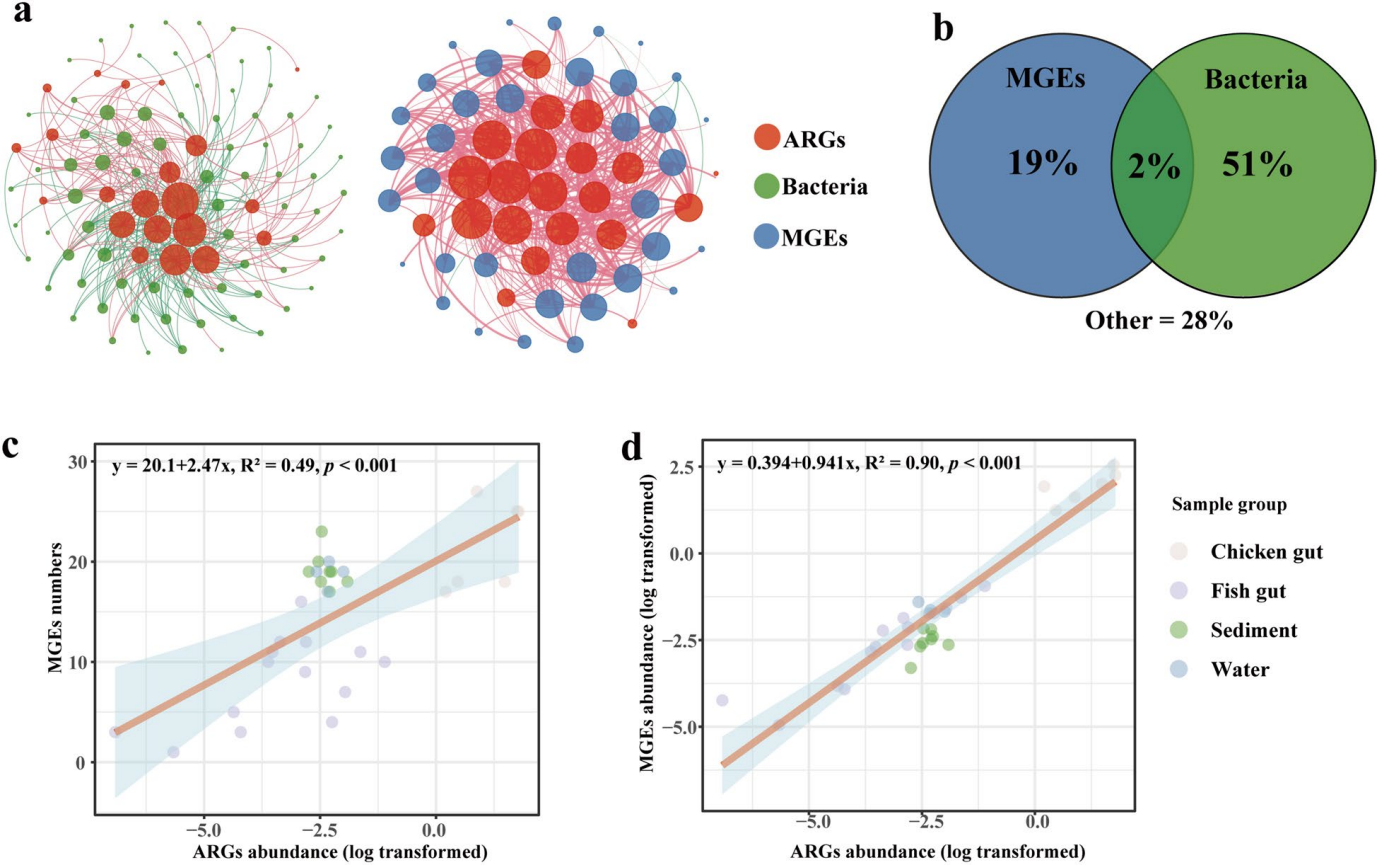
Influencing factors of ARGs in aquaculture system. **a** The network analysis shows the relationship between ARGs, MGEs, and bacteria (genus level) (*p* < 0.05). Larger nodes indicate more connections. The red lines indicate positive correlations, and the green lines indicate negative correlations. **b** VPA shows the interpretation rate of ARGs by MGEs and bacteria. **c** Linear relationship between ARG abundance and the number of MGEs (*p* < 0.001). **d** Linear relationship between ARG abundance and MGE abundance (*p* < 0.001) (discrete sample points f3, f4, and f15 were excluded)

Several results revealed the aquaculture ARGs were shaped by microbial communities and MGEs. Firstly, the abundance of ARGs was significantly correlated with the number (Fig. 3c) and abundance (Fig. 3d) of MGEs. Secondly, network analysis was used to explore the associations between ARGs, MGEs, and potential hosts (Fig. 3a). Seventy-seven genera and 36 MGEs were significantly associated with 23 ARG types (*p* < 0.05). The detail information of network analysis results can be found in Table S6. The results indicated that *Anabaena*, *Aphanizomenon*, *Cylindrospermopsis*, and *Raphidiopsis* were significantly correlated with 13 ARGs. *Fusobacterium* was observed to be associated with 8 ARGs. The insertion sequence (IS) was the dominant MGE type associated with ARGs, accounting for approximately 75% of all MGE types. Tn*916* and *qacEdelta* were associated with 16 and 13 ARGs, respectively. Aminoglycoside, sulfonamide, and fosmidomycin resistance genes were associated with the largest number of genera and MGEs. These ARG potential hosts and the correlation between ARGs and MGEs were also observed in analysis based on contigs and MAGs (refer to results 3.4 and 3.5). Finally, the impacts of MGEs and bacteria on ARGs were further quantitatively characterized by VPA (Fig. 3b). The VPA results indicated that bacterial flora could explain 51% of ARGs profile and MGEs could explain the other 19%.

In addition, Spearman’s rank coefficient revealed the correlation of core ARGs with MGEs and genera (Table S7). There was a relatively strong correlation between *Thiobacillus* with MLS resistance gene *mac*B and multidrug transporter (*p* ≤ 0.01). Many types of IS (ISSfl3, IS679, and IS621) were significantly positively correlated with *mac*B, multidrug transporter, and cAMP-regulatory protein (*p* < 0.01). The positive correlation was also identified between transposon *tni*A and *mac*B. The above evidence suggested that aquaculture core ARGs might be influenced by MGEs and bacteria.

### Co-occurrence of diverse ARG and MGE combinations

**Fig. 4:**
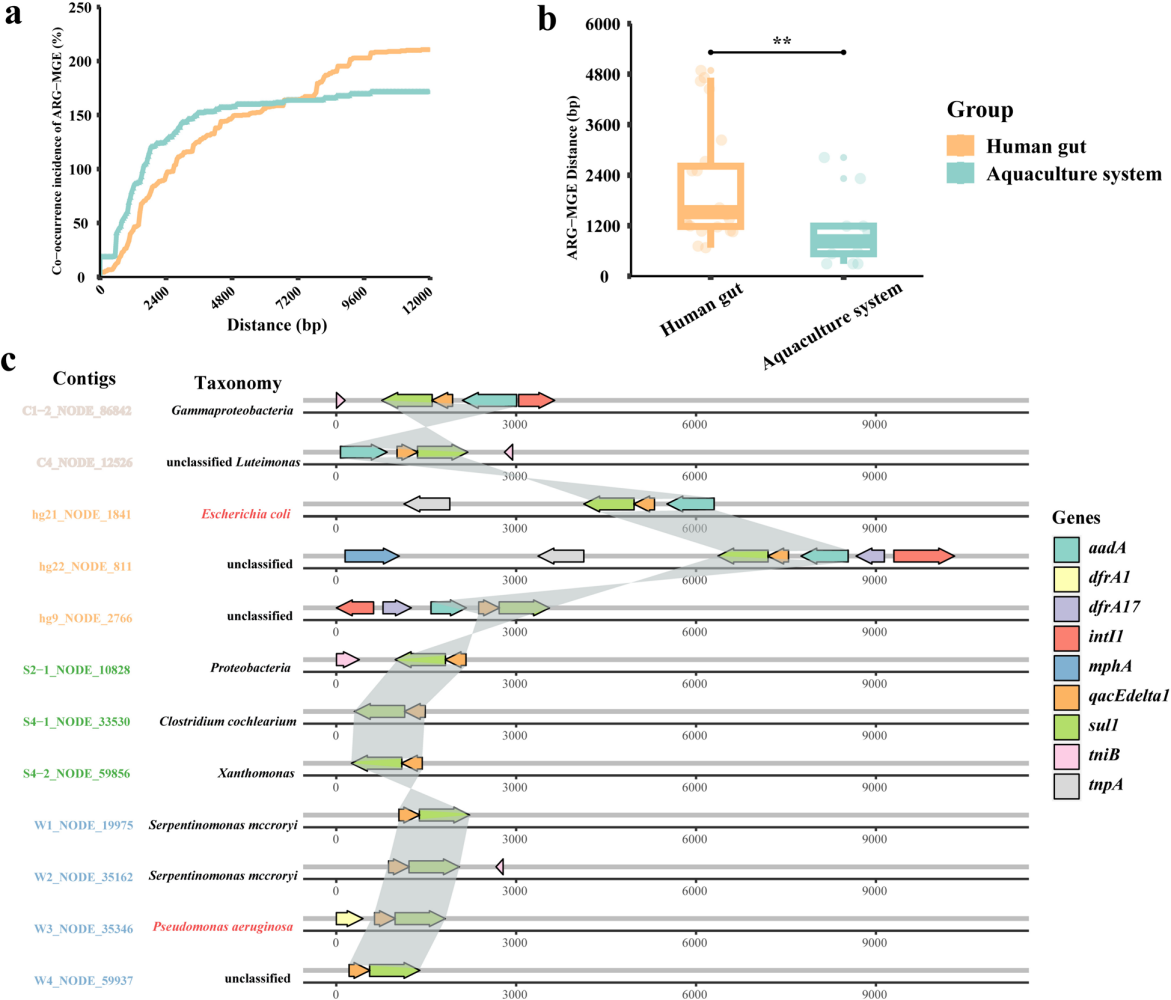
The co-occurrence pattern of ARGs and MGEs based on contigs. **a** The co-occurrence incidence of ARGs and MGEs. The *x*-axis means the distance of ARG-MGE, and the *y*-axis means the co-occurrence incidence. With the increase of distance, the co-occurrence rate also increases and gradually reaches a peak. **b** The shortest distance of ARG-MGE in the human gut and aquaculture system (**p < 0.01 indicates significant difference). **c** Gene arrangement patterns of *sul*1-*qacEdelta*1 and neighborhood genes in different sample groups. Different colored contigs belong to different sample groups, and the taxonomy names colored in red indicate opportunistic pathogenic bacteria

The transfer risk of ARGs in aquaculture systems was assessed by the incidence of co-occurrence and average minimum distance between ARGs and MGEs [52]. As the distance from ARGs increased, the incidence of MGE co-occurrence increased (Fig. 4a), and the distance between ARGs and MGEs in aquaculture system was significantly shorter than that in the human gut (*p* < 0.001) (Fig. 4b). In order to further reveal the co-occurrence pattern of ARGs and MGEs, we analyzed genetic locations of the two elements on metagenomic-assembled contigs retrieved from all samples. Overall, 71 ACCs were identified, among which 53 ACCs (75%) carried MGEs, 21 ACCs (30%) carried multiple ARGs, and 10 ACCs (14%) carried multiple ARGs and MGEs simultaneously. Furthermore, the co-occurrence of ARGs and MGEs shared by aquaculture systems and human gut samples were investigated, and various co-occurrence patterns of ARGs and MGEs were observed. The aminoglycoside resistance gene *aadA*, sulfonamide resistance gene *sul*1, and the quaternary ammonium compound (qac) resistance gene *qacEdelta*1 form a stable combination and were present in different sample groups (Fig. 4c). The *qac* gene was reported as a potential ancestor of class I integrons, and clinical class I integrons may contain *qacEdelta* gene [59, 60], hence the occurrence with the elements will increase the HGT probability of *aadA* and *sul*1. It is noteworthy that we identified two contigs W3_NODE_35346 and hg21_NODE_1841, predicted to be derived from opportunistic pathogens *Pseudomonas aeruginosa* and *Escherichia coli*, respectively.

In addition to the combination of *aad*A*-sul*1-*qacEdelta*1, multiple ARGs and MGEs were found to co-occur on various contigs from different samples, such as *sul*4-*tnp*A, *tet*A-*tnp*A, and *tet*M-Tn*916* (Fig. S2). Especially, the tetracycline resistance gene *tet*P, one of the core ARGs, was found to be widely distributed in chicken, fish, and human gut samples (Fig. S2c). The co-occurrence of another core ARG transcriptional regulatory protein *Cpx*R between integrase *int*2 and transposase *tnp*A was also observed in chicken and human gut samples.

### The source tracking of ARGs in the human gut

To further track the source of human gut ARGs, the Bayesian-based tool SourceTracker was used, and different environment types were set as potential sources. Four hundred fifty-seven metagenomic data collected from definite environments (including non-intensive aquaculture environments from this study) were used for analysis by a leave-one-out strategy to compare predicted environmental types with definite environmental types. The results showed that all environment types were predicted with more than 80% accuracy by five independent tests (Fig. S3). The aquaculture environment type was predicted with 88% accuracy comparable to previous studies [54]. The results indicated that SourceTracker could effectively predict ARG sources from different environment types. Then, the model was used to predict the potential source of ARGs in the human gut by five independent analyses. The result showed that the aquaculture environment was the main potential source of human gut ARGs, accounting for 81% (Table S8). This result identified the potential risk of ARG transfer from the aquaculture system to the human gut.

### Identification and quantification of ARG-carrying genomes (ACGs)

**Fig. 5:**
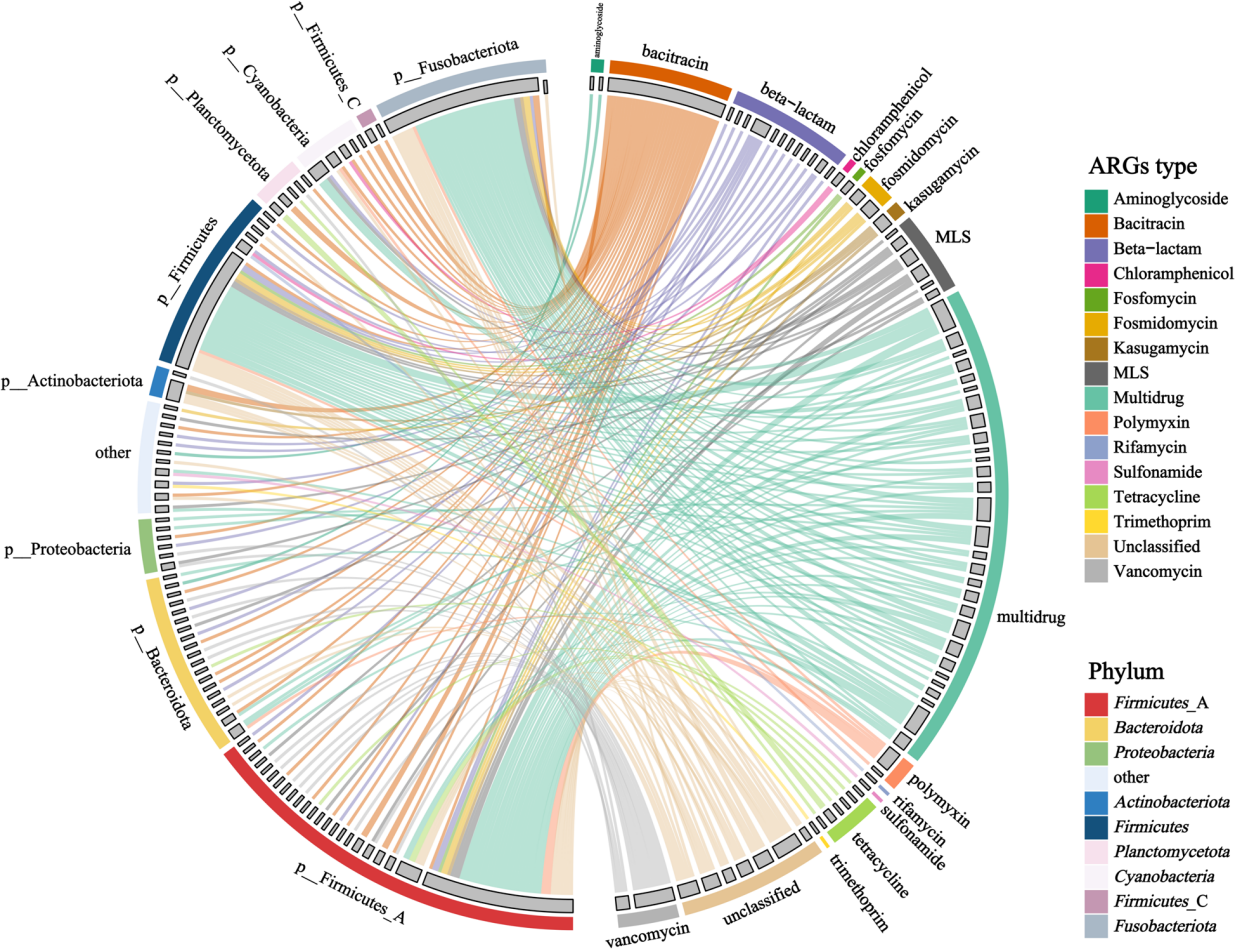
Circos diagram shows the ARGs carried by different MAGs. The outermost circle represents the MAG phylum level and ARG types, the gray rectangle represents the number of MAGs and ARG subtypes, and the innermost connecting lines are colored according to the ARG types

Recovering MAGs and detecting ACGs could provide insights into the host of ARGs and assess potential transfer risks. In total, 475 high-quality MAGs were recovered and the taxonomy of 460 non-redundant MAGs was annotated (Table S9). At the phylum level, the most abundant bacteria were *Firmicutes*, *Bacteroidota*, and *Proteobacteria*, and 12 bins were identified as opportunistic pathogenic bacteria (Fig. S4). The Circos plot displayed the distribution of ARGs across MAGs (Fig. 5), with MDR and bacitracin resistance genes being the two most abundant ARGs carried by MAGs. It is noteworthy that the MDR carried by MAGs contained 29 subtypes, while the bacitracin resistance genes contained only one subtype, *bac*A.

**Fig. 6:**
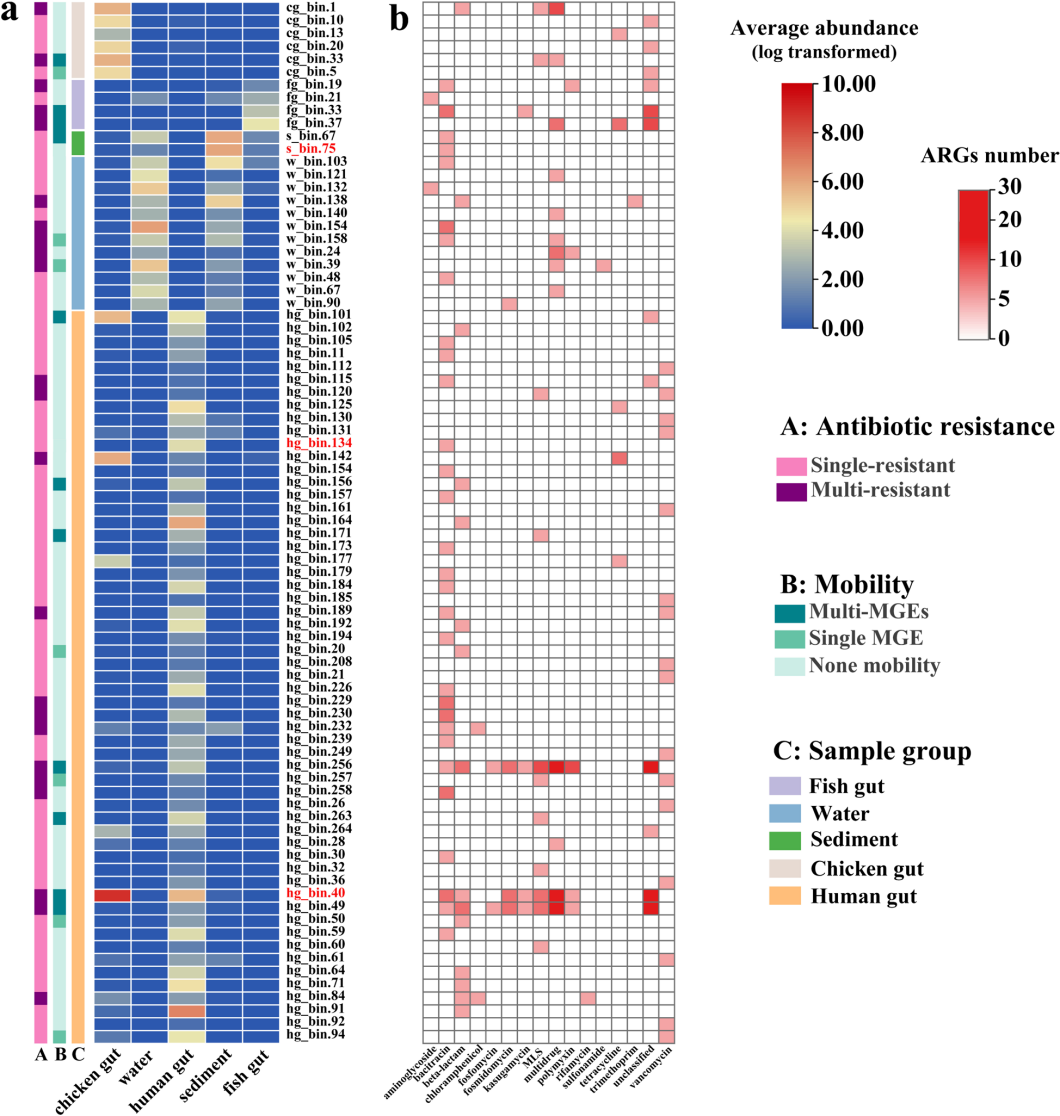
The heatmap shows the ACG abundance, mobility, and carried ARG types. **a** The color bar A indicates the ACG carries one or more ARGs. The color bar B indicates the ACG does not carry MGE, carries one MGE, or carries multiple MGEs. The color bar C indicates the sample group from which the ACG was recovered. The heatmap shows the average abundance of ACGs in different sample groups (data was log transformed). ACG name labels in red indicate opportunistic pathogenic bacteria. **b** The number of ARG types carried by ACGs. The color indicated the carried ARG number

The heatmap further displayed the relative average abundances of ACGs in different sample groups and the types of ARGs carried (Fig. 6). Strikingly, some ACGs carried multiple ARGs which may be highly mobile. For all 81 ACGs, 23 carried multiple ARGs, 18 carried MGEs, and 6 ACGs carried multiple ARGs and MGEs simultaneously (Table S10), which were considered as genomes with high ARG enrichment and mobility, thus posing a high risk of promoting ARG dissemination. These 6 ACGs were hg_bin.40 (*Fusobacterium*_A sp900543175), hg_bin.256 (*Eubacterium*_G sp000434315), hg_bin.49 (*Holdemanella* porci), fg_bin.37 (*Acetatifactor* intestinalis), fg_bin.33 (*Solirubrobacterales* bacterium 67–14), and cg_bin.33 (*Nannocystaceae*) carrying 48, 46, 38, 7, 6, and 2 ARGs, respectively, which encode bacitracin, beta-lactam, fosfomycin, fosmidomycin, kasugamycin, MLS, MDR, polymyxin, and tetracycline resistance.

Especially, 11 MAGs carrying core ARGs were identified (Table S11). Five MAGs carried multiple core ARGs and 7 MAGs also carried MGEs, while no MAG carrying core ARGs were identified as opportunistic pathogens. It is worth noting that hg_bin.40 carried MGEs and 4 core ARGs simultaneously, and the relative abundances of hg_bin.40 was high in both chicken gut and human gut samples (Fig. 6a), suggesting the potential risk of core-ARG transfer.

## Discussion

A non-intensive aquaculture farm was selected for sampling to fill the research gap on non-intensive aquaculture resistomes. A study about the large-scale intensive aquaculture farms in Guangdong Province found that the concentrations of erythromycin in fishpond water could reach 80–1400 ng/L [16], much higher than this study (< 1 ng/L, Table S2). Despite the lack of antibiotic pressure in non-intensive aquaculture environment, multiple ARG types were also detected (Fig. 1a). It has been reported that MDR and bacitracin resistance genes were the main resistance types in freshwater aquaculture [20], which were similar to our findings (Fig. 1a). It is worth noting that although the average abundance of ARGs detected in this research was approximately 0.06 copies of ARG per copy of 16S rRNA gene (Fig. 1b), which was lower than 0.25 copies of ARG per copy of 16S rRNA gene in a previous study about integrated freshwater aquaculture farms [26], the potential transfer risk of ARGs could be still demonstrated (Fig. 4c). Despite almost zero use of antibiotics in this relatively closed non-intensive aquaculture system, fishpond irrigation water sourced from surrounding river may introduce extra antibiotic pressure. The relatively low concentrations of antibiotics and metals (Table S2) might form a combined selection pressure leading to the occurrence and transfer of ARGs [61]. These findings suggest that the mobility of ARGs needs more attention even in non-intensive aquaculture environments with low antibiotic pressure.

Previous studies have suggested that the microbial structure of fish gut was influenced by the host species and diet [62, 63]. However, the microbial structure of 5 different fish species in our research did not show significant differences (*R*^2^ = 0.33, *p* = 0.18) (Fig. S5a). The fish gut microbiome could profile the intestinal ARGs by affecting the hosts’ metabolism [64], while there was no significant difference between the intestinal ARGs of different fish species either (*R*^2^ = 0.23, *p* = 0.55) (Fig. S5b). This might be due to the fact that the water and feed received by the fish were from the same surrounding environments in our research, thus shaping the similar gut community and resistomes of different fish species. These results suggest that the fish gut microbiome and ARGs might be determined by the habitat rather than fish species [65–67]. Extensive large-scale research is needed to explore the contribution of habitat environments to fish gut resistomes in the future. Moreover, approximately 40% of the ARGs carried by the fish were identified as aquaculture core ARGs that indicated fish may play an important role in the transmission of aquaculture ARGs (Fig. 2c).

The core ARGs have been demonstrated to exist in a variety of environments [68–70]. To accurately identify core ARGs in aquaculture systems, more stringent criteria (approximately the top 3% most abundant with detection frequency greater than 80% in all aquaculture system samples) were used to define aquaculture core ARGs compared to previous studies [69, 71]. The results showed that 33% of core ARGs in aquaculture environments were MDR, similar to the composition of core ARGs in municipal wastewater treatment plants [72, 73] and farmland soils [71]. Notably, only a small fraction of the human gut ARG profile consisted of the core ARGs (4% ± 2%). Previous research indicated that the HGT of core ARGs was seldom observed due to the barriers of bacterial phylogeny and the absence of MGEs [74, 75]. However, the core ARGs were highly shared between different aquaculture system samples (29% ± 20%, Fig. 2c), and one of the aquaculture core ARG *tet*P was shared between the aquaculture system and human gut (Fig. S2). In this work, different samples were collected from a relatively closed aquaculture system, circulation of materials, and microbiome transfer could be frequent, which might increase the possibility of core ARGs exchange between different environmental components.

Those ARGs capable of transferring from the environment to human gut are of high health risks, which call for priority attention when conducting relevant surveillance and risk assessment [76]. In this study, 262 ARG subtypes were co-shared by aquaculture system and human gut (Fig. 2b). ARGs in aquaculture system had a positive correlation with MGEs indicating the potential mobility of aquaculture ARGs (Fig. 3c and d). The gene arrangement showed the possible transfer of different ARG-MGE combinations between aquaculture environment and human. In addition, some MGEs (*int*I1, *tni*B, and *tnp*A) were located in proximity of ARGs, suggesting the high mobility of these ARGs (Fig. 4c). The relatively short distance between ARGs and MGEs implied high risk of ARGs transfer, of which the mobility of those clinical important ARGs between aquaculture environment and human may raise public health concern (Fig. 4b). Furthermore, the results of source tracking showed that aquaculture might occupied 80% of the ARG contribution in the human gut (Table S8), which further indicated the potential risk of ARG transfer from aquaculture environment to the human gut. In fact, it has been reported that the resistomes could be shared between human and animal gut [9, 77–79]; however, there is limited evidence of resistomes transmission from environment to human due to factors like ecological barrier, phylogenetic restriction, and low mobility [74, 75]. The combination of *sul*1-*qacEdelta*1, which could transfer from aquaculture system to human gut, has not only been detected in this study (Fig. 4c), but also been reported in wastewater treatment plants [80], plastisphere [81], and drinking water [82]. These results provide the evidence for resistomes transfer potential between environments and humans, thus indicate that the high mobility of aquaculture resistomes, especially the ARG transfer from environmental bacteria to clinical pathogens raise concern of infectious untreatable [76, 83]. Furthermore, samples based on time series will help explore the specific transfer process of resistomes between the environment and human in the future.

Identification and quantification of human opportunistic pathogenic bacteria could assist in assessing relevant health risks [84]. In this study, contig hg21_NODE_1841 obtained from human gut was identified as *E. coli* and W3_NODE_35346 obtained from fishpond water was identified as *P. aeruginosa* (Fig. 4c). These contigs carried ARG-MGE and were recognized as human opportunistic pathogenic bacteria meanwhile indicating the potential pathogenicity of aquaculture resistomes. Additionally, 12 opportunistic pathogenic MAGs were obtained and 75% of them were recovered from human gut (Table S9). It is worth noting that high abundances of hg_bin.40 (*Fusobacterium*) were detected in both chicken gut and human gut (Fig. 6a). *Fusobacterium* species have been found to cause a wide range of opportunistic infections related to oral diseases and colorectal carcinoma [85]. The *Fusobacterium* MAG was found carried multiple ARGs and MGEs, which was similar to a previous study that *Fusobacterium* might carry tetracycline resistance genes [86]. The detection of same opportunistic pathogenic bacteria both in human gut and aquaculture system samples revealed the potential role for aquaculture in promoting pathogenicity of human gut microbiome. In fact, only 3% MAGs were detected as opportunistic pathogenic bacteria and 1% MAGs carried multiple ARGs and MGEs in this study. Compared to culture-based experiments, MAG-based methods are limited in only capturing those abundant bacteria in sequencing, providing indirect evidence about ARG mobility or bacterial pathogenicity [87], lacking phenotypic characteristics [77]. In the future, transcriptome data and culture-based experiments are needed to complement the metagenomics in providing a more comprehensive insight into the risks of ARGs and the host microorganisms.

## Conclusion

This research explores the transmission capability of resistomes and potential risks to human health in the understudied non-intensive aquaculture environment. Multiple types of ARGs were identified with possible mobility in the aquaculture environment. The detected ARG-MGE combinations had potential transfer risk between human gut and aquaculture system. The potential transfer of opportunistic pathogens carrying multiple ARGs between aquaculture system and human gut could increase the risk of human disease. In summary, our study emphasizes the aquaculture environment even with almost zero antibiotic application as “hidden” reservoir of ARGs and indicates potential mobility of resistomes across human–environment boundaries.

### Supplementary Information

**Additional file 1: Fig. S1.** The relationship between the mean number and abundance of detected ARGs with the occurrence frequency. **Fig. S2.** Gene arrangements of ARG-MGE combinations. **Fig. S3.** The result of source tracking by leave-one-out strategy. **Fig. S4.** The phylogenetic tree of MAGs. **Fig. S5.** The PCoA of fish gut microbiome and ARGs. ARGs: antibiotic resistance genes. MGEs: mobile genetic elements. MAGs: metagenomic-assembled genomes. PCoA: principal coordinate analysis.

**Additional file 2: Table S1.** The composition of the fish feed. **Table S2.** The concentrations of antibiotics and metals in fishpond water. **Table S3.** The detailed sample information. **Table S4.** The information of human gut samples. **Table S5.** The information of different environment samples used for source tracking. **Table S6.** The specific network association between ARGs, MGEs and genera. **Table S7.** The Spearman’s rank correlation between core ARGs, MGEs and genera. **Table S8.** The source tracking results of human gut ARGs. **Table S9.** The information of 460 non-redundant MAGs. **Table S10.** The detailed information of 6 ACGs carried multiple ARGs and MGEs. **Table S11.** The detailed information of 11 MAGs carrying core ARGs.

## Data Availability

All sequencing data is available at the Sequence Read Archive (SRA) of NCBI (PRJNA988937).
