# Supplementary material for: Metagenomic approach revealed the mobility and co-occurrence of antibiotic resistomes between non-intensive aquaculture environment and human
Source: Microbiome. 2024 Jun 14;12:107. doi: 10.1186/s40168-024-01824-x (PMC11179227; doi:10.1186/s40168-024-01824-x)
Supplement: Supplementary file 2 — Additional file 1: Fig. S1. The relationship between the mean number and abundance of detected ARGs with the occurrence frequency. Fig. S2. Gene arrangements of ARG-MGE combinations. Fig. S3. The result of source tracking by leave-one-out strategy. Fig. S4. The phylogenetic tree of MAGs. Fig. S5. The PCoA of fish gut microbiome and ARGs. ARGs: antibiotic resistance genes. MGEs: mobile genetic elements. MAGs: metagenomic-assembled genomes. PCoA: principal coordinate analysis. [file 40168_2024_1824_MOESM1_ESM.docx]

**Supplementary Information**

Metagenomic approach revealed the mobility and co-occurrence of antibiotic resistomes between non-intensive aquaculture environment and human

Li Tian^a^, Guimei Fang^a^, Guijie Li^a^, Liguan Li^b, c^, Tong Zhang^b, c^, Yanping Mao^a,*^

^a^ College of Chemistry and Environmental Engineering, Shenzhen University, Shenzhen, 518071, Guangdong, China

^b^ The University of Hong Kong Shenzhen Institute of Research and Innovation, Shenzhen, 518057, Guangdong, China

^c^ Environmental Microbiome Engineering and Biotechnology Laboratory, Centre for Environmental Engineering Research, Department of Civil Engineering, The University of Hong Kong, Hong Kong SAR, China

*Corresponding author: Tel: +86-26558094; E-mail address: [maoy@szu.edu.cn](mailto:maoy@szu.edu.cn) (Y. Mao)

# Supplementary Figures

## **Fig. S1** The relationship between the mean number and abundance of detected ARGs with the occurrence frequency.

## **Fig. S2** Gene arrangements of ARG-MGE combinations.

## **Fig. S3** The result of source tracking by leave-one-out strategy.

## **Fig. S4** The phylogenetic tree of MAGs.

## **Fig. S5** The PCoA of fish gut microbiome and ARGs.

ARGs: antibiotic resistance genes.

MGEs: mobile genetic elements.

MAGs: metagenomic-assembled genomes.

PCoA: principal coordinate analysis.

# Supplementary Figures


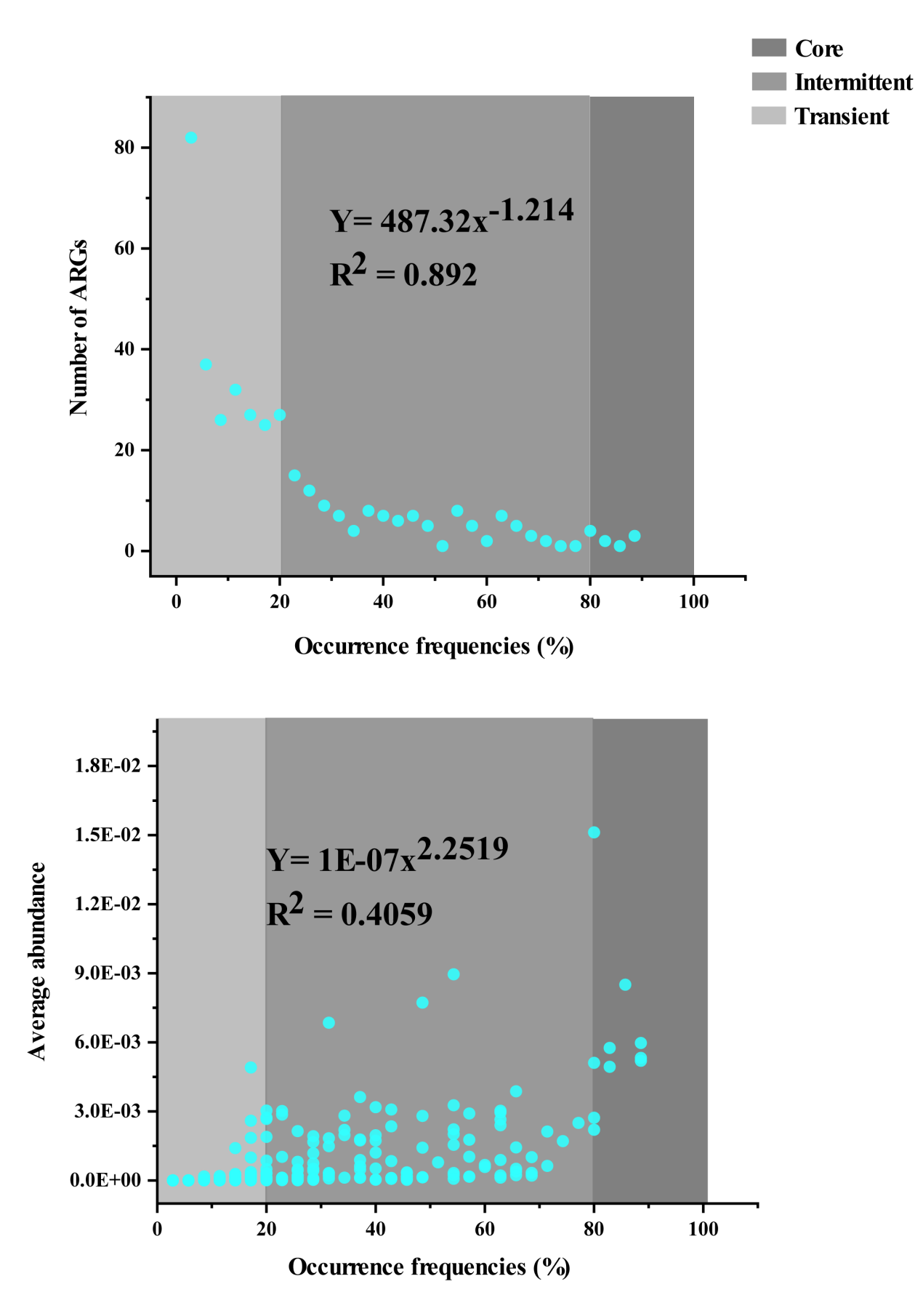


**Fig. S1** The relationship between the mean number and abundance of ARGs with the occurrence frequency.


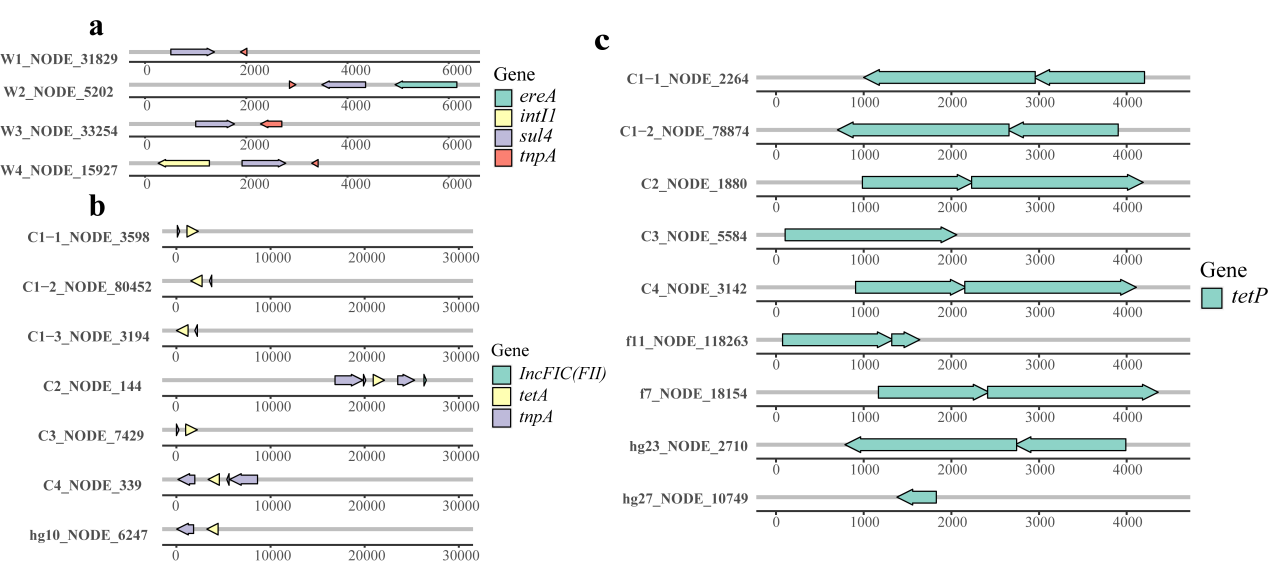


## **Fig. S2** Gene arrangements of ARG-MGE combinations. a: *sul*4 in water samples. b: *tet*A-*tnp*A in chicken gut and human gut samples. c: *tet*P in aquaculture system and human gut.


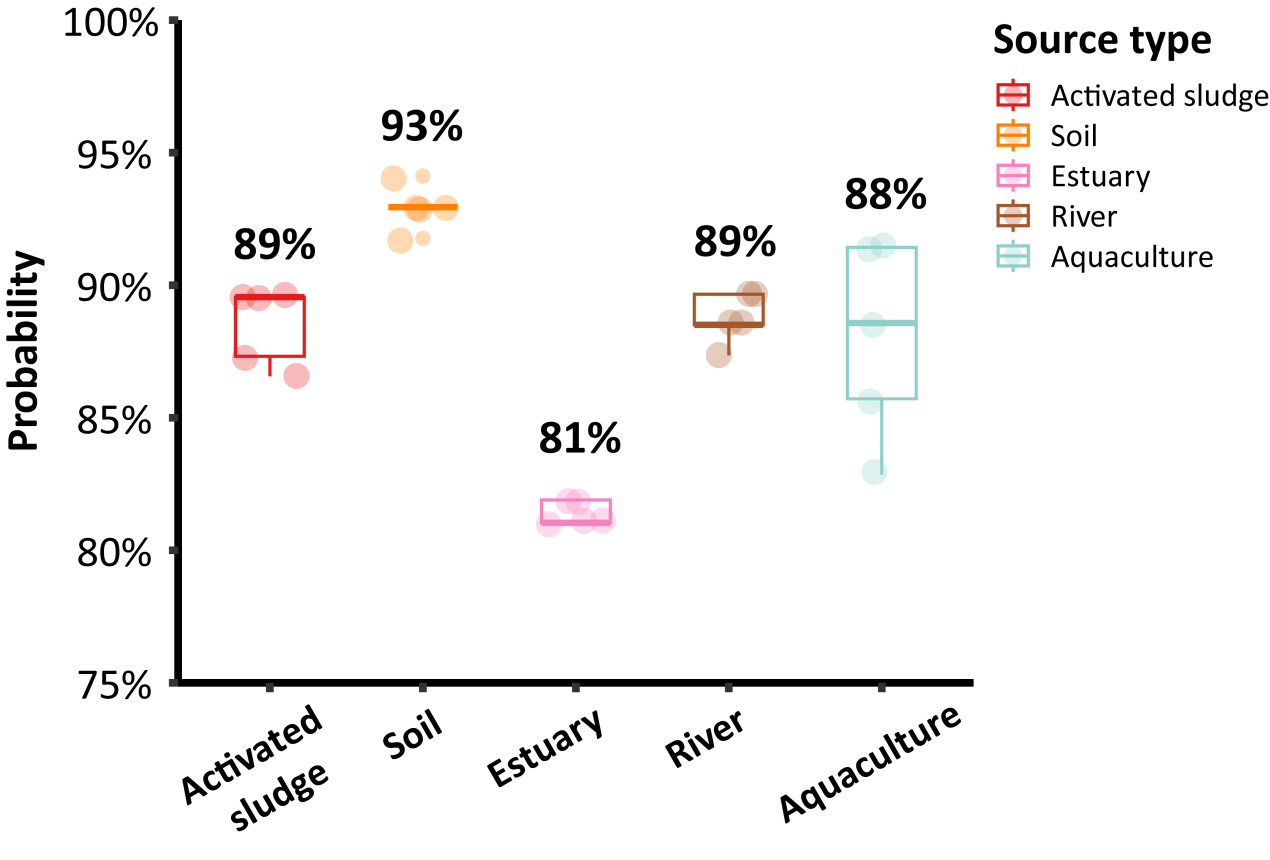


**Fig. S3** The result of source tracking by leave-one-out strategy. The X-axis represents the different types of environmental sources. The Y-axis shows the proportion of correct predictions by SourceTracker.

## **
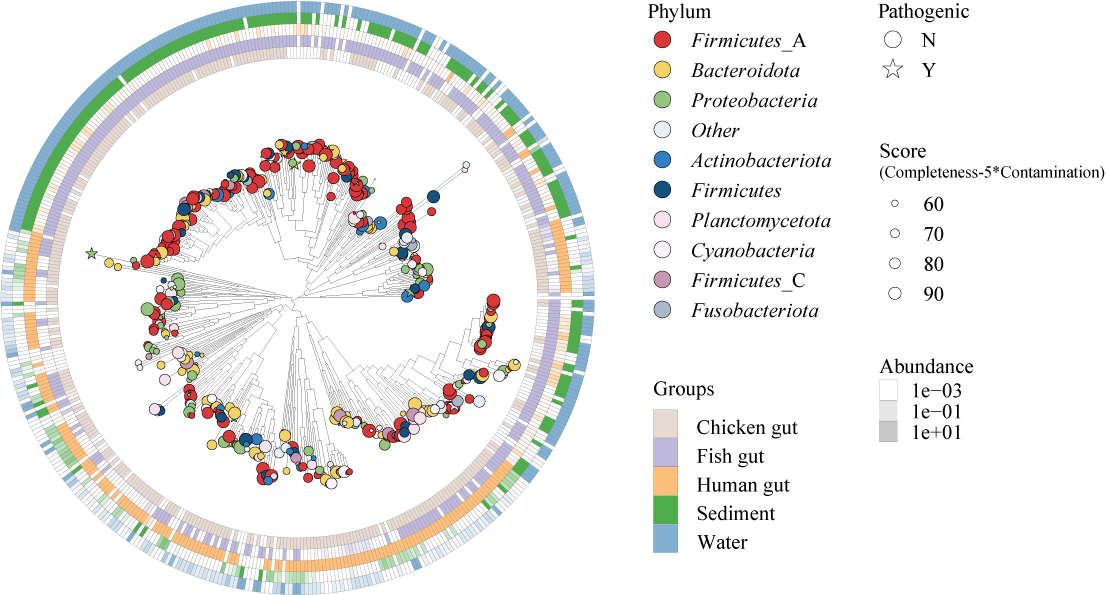
**

## **Fig. S4** The phylogenetic tree of MAGs. The heat map of the outermost ring shows the abundance of MAGs (colored according to sample groups). The size of the inner points indicates the quality of the MAG, and the points are stained according to different phyla. Pathogenic bacteria are represented by the shape of star.


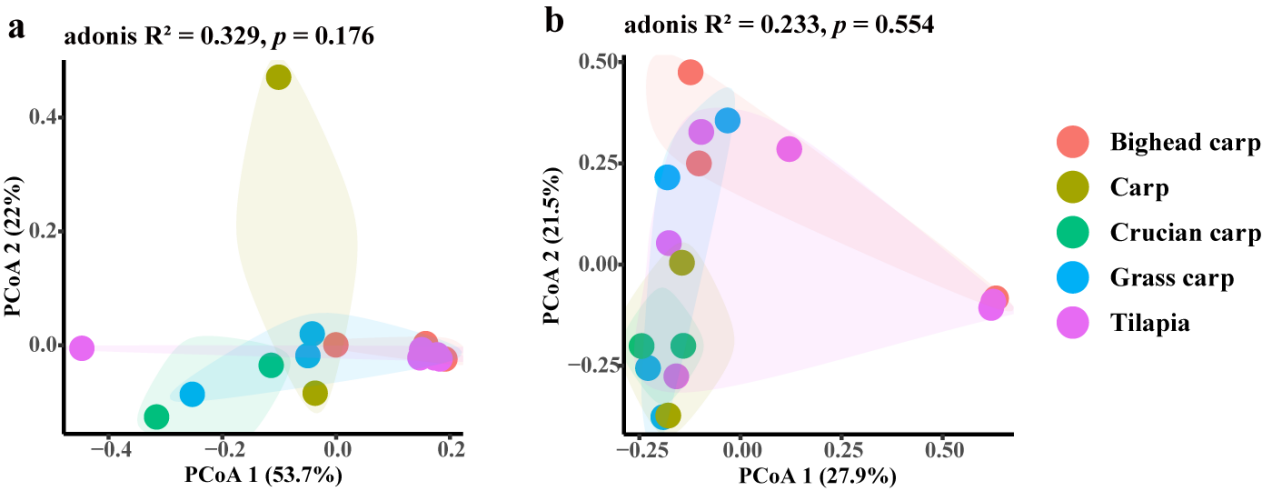


## **Fig. S5** The PCoA of fish gut microbiome and ARGs. a: PCoA of fish gut genus. b: PCoA of fish gut ARGs.
